# Impacts of genomic alterations on the efficacy of HER2-targeted antibody–drug conjugates in patients with metastatic breast cancer

**DOI:** 10.1186/s12967-025-06082-5

**Published:** 2025-01-13

**Authors:** Riqing Huang, Anqi Hu, Qixiang Rong, Ditian Shu, Meiting Chen, Wei Yang, Yue Zhang, Qiufan Zheng, Xin An, Cong Xue, Haifeng Li, Yanxia Shi

**Affiliations:** 1 State Key Laboratory of Oncology in South China, Guangdong Provincial Clinical Research Center for Cancer, Sun Yat-Sen University Cancer Center, No.651 Dongfeng East Road, Guangzhou, 510060 People’s Republic of China; 2 Department of Medical Oncology, Sun Yat-Sen University Cancer Center, No.651 Dongfeng East Road, Guangzhou, 510060 People’s Republic of China

**Keywords:** Breast cancer, Antibody–drug conjugate, Cell cycle, CDK12

## Abstract

**Background:**

HER2-targeted antibody–drug conjugates (ADCs) have revolutionized the treatment landscape of metastatic breast cancer. However, the efficacy of these therapies may be compromised by genomic alterations. Hence, this study aims to identify factors predicting sensitivity to HER2 ADC in metastatic breast cancer.

**Methods:**

This comprehensive real-world retrospective study collected clinical data from patients diagnosed with metastatic breast cancer and performed genomic profiling using targeted next-generation sequencing. The study analyzed the associations between genomic alterations and clinical outcomes of HER2 ADC treatment.

**Results:**

Sixty-three patients were included in this study, 33 with HER2-low breast cancer and 30 with HER2-positive breast cancer, respectively. The most frequently altered genes were *TP53* (69%), *PIK3CA* (45%), *MYC* (35%), and *ERBB2* (35%). Patients with amplifications in cell cycle-related genes showed inferior median progression-free survival (PFS) than those without amplifications (2.07 months vs. 8.40 months; HR = 5.24; 95% CI 2.11–13.01; *p* < 0.001), particularly in HER2-low patients (2.07 months vs. 8.27 months; HR = 4.23; 95% CI 1.50–11.91; *p* = 0.004). Additionally, *ERBB2/CDK12* co-amplification exhibited a superior median PFS in all patients (19.33 months vs. 5.43 months; HR = 0.13; 95% CI 0.04–0.45; *p* < 0.001) and in HER2-positive patients (19.33 months vs. 6.87 months; HR = 0.18; 95% CI 0.05–0.72; *p* = 0.007). Multivariate analysis indicated that amplification in cell cycle-related genes was an independent predictor of inferior PFS (HR = 4.46; 95% CI 1.08–18.40; *p* = 0.039), while the presence of *ERBB2/CDK12* co-amplification was independently correlated with superior PFS (HR = 0.16; 95% CI 0.04–0.65; *p* = 0.010).

**Conclusions:**

Amplification in cell cycle genes may contribute to primary resistance of HER2 ADC in HER2-low breast cancer. *ERBB2/CDK12* co-amplification may be a potential biomarker for favorable responses in HER2-positive breast cancer.

**Supplementary Information:**

The online version contains supplementary material available at 10.1186/s12967-025-06082-5.

## Introduction

Breast cancer represents one of the most common malignancies among women worldwide [1], with metastatic breast cancer (MBC) being a leading cause of cancer-related mortality. The therapeutic landscape for MBC has evolved significantly with the introduction of antibody–drug conjugates (ADCs). These advanced treatments combine the targeted precision of monoclonal antibodies with the potent cytotoxicity of chemotherapeutic agents, enhancing drug delivery to HER2-overexpressing tumor cells, thereby maximizing therapeutic efficacy while minimizing off-target effects. Although these ADCs provide some clinical benefit in patients with HER2-overexpressing and HER2-low MBC, most will ultimately experience disease progression and die. Despite these advancements, the field lacks reliable predictive biomarkers for therapeutic efficacy. Furthermore, the mechanisms underlying resistance to these treatments remain largely undefined, presenting significant challenges in managing non-responsive patients. These issues underscore the urgent need for focused research to identify both predictive biomarkers and resistance mechanisms, which could substantially enhance the precision and effectiveness of ADCs for MBC.

Recent research has increasingly focused on genomic profiles to identify potential predictors of response and resistance. Preliminary studies have started to map the genetic alterations that could influence the effectiveness of HER2-targeted therapies. An exploratory analysis of the phase 2 DAISY trial in breast cancer reported that HER2 hemizygous deletion and SLX4 mutations likely underlie primary acquired resistance to trastuzumab deruxtecan (T-Dxd) [2]. Translational research involving 20 HER2-amplified patients from a Phase I study confirmed that the co-amplification of CDK12, compared to no co-amplification, is a promising biomarker for predicting a better response to KN026, a novel bispecific antibody that simultaneously binds to two distinct HER2 epitopes [3]. Loss of HER2 amplification in tumor cells has been observed in circulating tumor DNA from a small cohort of patients and was found to be associated with trastuzumab emtansine (T-DM1) resistance [4]. However, comprehensive genetic profiling in this context is still not widespread, and the data are yet to be systematically integrated into clinical practice.

A comprehensive understanding of the mechanisms of action and resistance may enhance treatment selection for patients and facilitate the development of more effective therapeutic strategies. Recognizing the complexity and heterogeneity of MBC, we have initiated a real-world study to explore how specific genomic profiles correlate with the response to HER2-targeted ADC therapy. This study aims to collect and analyze comprehensive genomic and clinical data from a diverse patient cohort, providing a robust foundation for identifying predictive biomarkers and resistance mechanisms. By integrating genomic insights with clinical outcomes, this research seeks to refine predictive models for treatment response and guide the development of next-generation targeted therapies for metastatic breast cancer.

## Materials and methods

### Study design and patients

This retrospective real-world study utilized clinical and genomic data from patients with metastatic breast cancer who received HER2 ADCs at the Sun Yat-sen University Cancer Centre (SYSUCC) between Jan 23, 2018, and May 23, 2024. The inclusion criteria were: 1) histologically confirmed breast cancer with metastatic disease at enrollment, 2) treatment with HER2 ADCs, including trastuzumab emtansine (T-DM1), trastuzumab deruxtecan (T-DXd), and disitamab vedotin (RC48), 3) undergone genomic profiling, and 4) complete clinical profiles.

### Data collection and definitions

The data were extracted from the medical charts and included the patients’ demographics, tumor characteristics, treatment, standard laboratory tests, and image scans. Generally, the patients were treated with each kind of ADC until disease progression, intolerable toxicity, or death based on the physicians’ experience. The dosage of ADCs was given basically according to their instructions.

HER2 expression was mainly detected by immunohistochemistry (IHC). The IHC scores were assessed according to the HER2 test guidelines for breast cancer [5]. The HER2 gene amplification could also be evaluated by fluorescence in situ hybridization (FISH), which compliant with the HER2 test guidelines for breast cancer [5]. Her-2 low status was defined as IHC 1 + or IHC 2 + and FISH negative. Hormone receptor-positive disease was defined as immunoreactive for estrogen or progesterone receptors in ≥ 1% of tumor-cell nuclei according to local testing.

The objective response was a response sustained for a minimum of two consecutive imaging evaluations at least 4 weeks apart. The disease was evaluated using RECIST version 1.1 for response assessment. Progression-free survival (PFS) was defined as the time from the initiation of treatment to progressive disease (PD) or death. Overall survival (OS) was measured from the initiation of treatment until the date of death. Follow-up CT scan data were collected for 2 years or until PD. Patients with PFS longer than 6 months were classified as good responders and patients with PFS shorter than 6 months were classified as poor responders.

### Targeted sequencing

Genomic testing encompassed hybrid capture-based targeted next-generation sequencing (NGS) to identify targetable alterations at the Molecular Diagnostics Department of SYSUCC. Initially, genomic DNA was extracted and sheared from archival formalin-fixed paraffin-embedded tumor along with matched normal tissues. cfDNA was detected and matched normal DNA from the control whole‐blood samples of the patient to determine somatic alterations of ctDNA. Subsequently, sequencing libraries were generated, ensuring a consistent median depth (> 500 ×), and assessed for somatic variants, including single nucleotide variants, small insertions and deletions, copy number alterations, and gene fusions/rearrangements.

### Statistical analysis

Statistical analyses and data visualization were performed utilizing R version 4.2.2(The R Project for Statistical Computing, www.r-project.org). Descriptive statistics (percentages and medians) were used to summarize patient characteristics, treatment administration, and antitumor activity. A cut-off date of June 25th, 2024, was established for analyzing data for this report. Hazard ratios (HR) and associated 95% confidence intervals (CIs) were calculated by Cox proportional hazards models. Kaplan–Meier survival plots were generated based on gene mutations and curves were compared using log-rank tests. All statistical tests performed in the present article were two-sided, and *p* values < 0.05 were considered significant.

## Results

The study included 63 patients, 33 with HER2-low breast cancer and 30 with HER2-positive breast cancer. Of the HER2 low cohort, 28 patients were luminal (HR-positive), and 5 patients were triple-negative breast cancer (TNBC). For the HER2 positive cohort, 12 patients were HR-positive, while 18 patients were HR-negative. The patients were aged from 30 to 76 years, with a median age of 50 years, 12 patients (19.05%) under 40 years old, and 9 (14.29%) over 65 years old. The majority (74.60%) presented with visceral disease. The most common metastasis included bone (53.97%), liver (46.03%), brain (28.57%), and lung (25.40%). Patients received HER2 ADCs approved by the Food and Drug Administration (FDA) or National Medical Products Administration of China, including T-Dxd (68.25%), RC48 (14.29%), and T-DM1(17.46%). The details of patients’ characters were summarized in Table 1.

**Table 1:** Patient characteristics

Characteristic	No. of patients (%)
Age	
Median (Range), years	50(30–76)
HR status	
Negative	23 (36.51)
Positive	40 (63.49)
HER2 IHC	
1 +	17 (26.98)
2 +	22 (34.92)
FISH +	6 (9.52)
FISH -	16 (25.40)
3 +	24 (38.10)
HER2 status	
HER2-positive	30 (47.62)
HER2-low	33 (52.38)
Histopathological	
Invasive ductal carcinoma	58 (92.06)
Invasive lobular carcinoma	2 (3.17)
Others	3 (4.76)
Presence of visceral disease	
Yes	47 (74.60)
No	16 (25.40)
Liver metastasis	
Yes	29 (46.03)
No	34 (53.97)
Brain metastasis	
Yes	18 (28.57)
No	45 (71.43)
Line of therapy	
1	4 (6.35)
2	15 (23.81)
3	13 (20.63)
≥ 4	31 (49.21)
Regimen	
T-DXd	43 (68.25)
RC48	9 (14.29)
T-DM1	11 (17.46)

**Fig.1:**
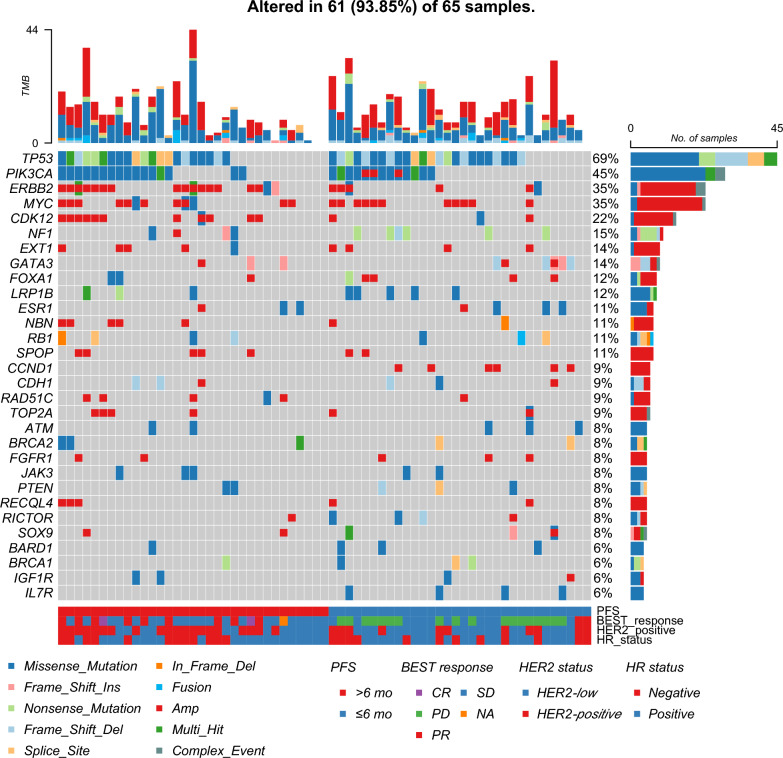
The landscape of high-frequency altered genes. Each column represents an individual patient. PFS, progression-free survival

IHC, immunohistochemistry; HR, hormone Receptor; FISH, fluorescence in situ hybridization;

The landscape oncoplot of genomic alterations for 65 samples with available NGS results of tumor samples was presented in Fig. 1. These genomic data were obtained from 53 FFPE and 12 blood samples, of which 2 patients had both FFPE and blood sample data. The most frequently altered genes were *TP53* (69%), *PIK3CA* (45%), *MYC* (35%), and *ERBB2* (35%). In the subsequent profiling, we found that three genes (*CCND1*, *ERBB2,* and *CDK12*) had different alteration trends between groups (all *p* < 0.05) (Table S1). Additionally, 14 patients had *ERBB2* amplification and 11 of them had *CDK12* co-amplification. According to Oncogenic Signaling Pathways in The Cancer Genome Atlas [6], which encompasses cell cycle, MYC, TP53, WNT, RTK-RAS, PI3K, and other signaling pathways, we observed that patients with amplifications in one or more cell cycle-related genes, including CCND1, CDKN1B, CDK4, CCND2, CCNE1, and CDK6, exhibited poor response (Fig.S1). The targeted gene panel is detailed in Table S2.

**Fig.2:**
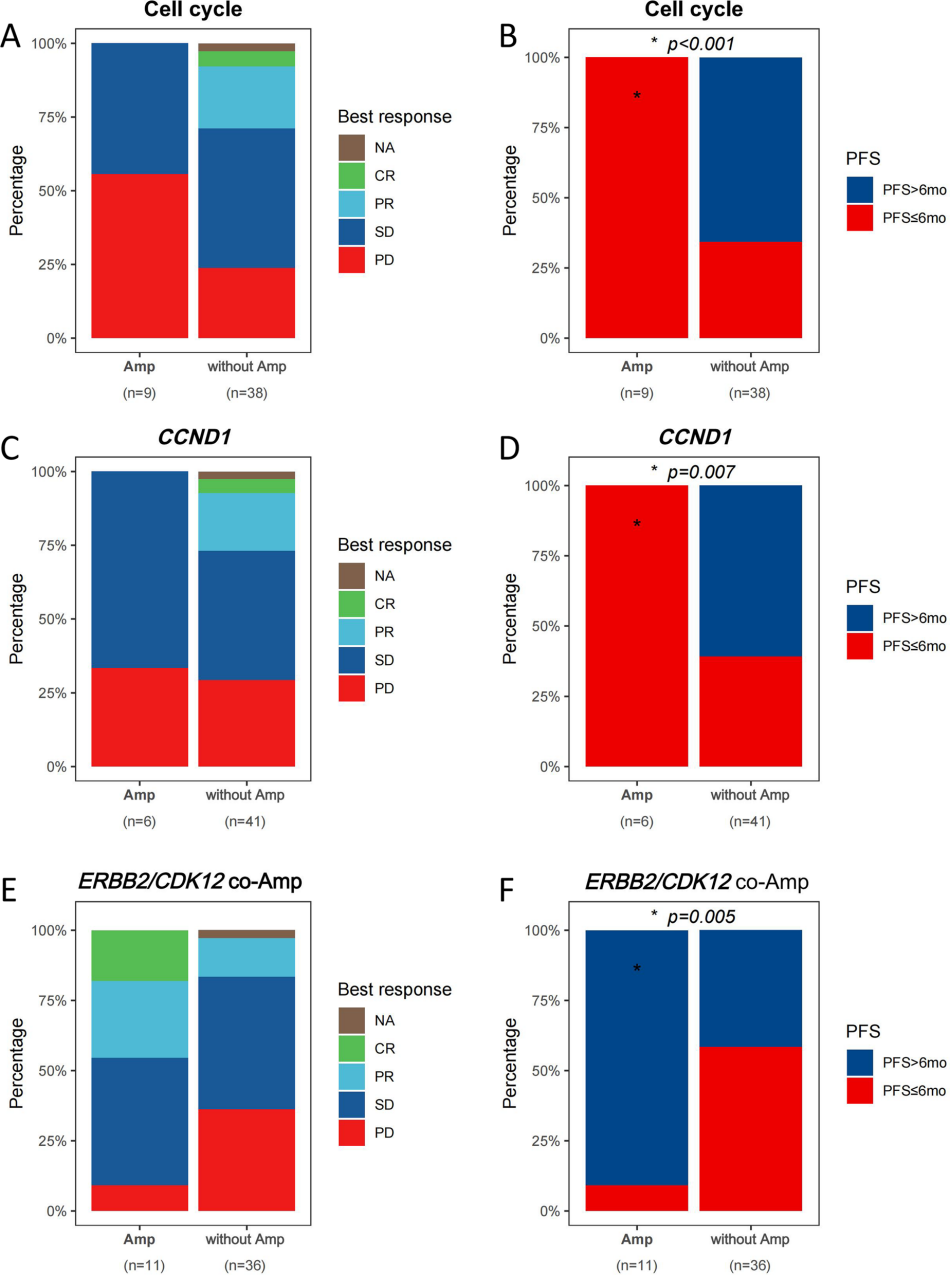
Distribution of treatment responses (**A**) and progression-free survival (PFS) (**B**) for patients with and without amplifications in cell cycle-related genes. Distribution of treatment responses (**C**) and PFS (**D**) for patients with and without *CCND1* amplification. Distribution of treatment responses (**E**) and PFS (**F**) for patients with and without *ERBB2/CDK12* co-amplifications

To investigate the associations between genomic alterations and clinical outcomes, 47 pretreatment specimens were used. We found that patients with amplifications (n = 9) in cell cycle-related genes exhibited distinctly poorer anti-tumor responses compared to those without amplifications (n = 38), with the best outcome being stable disease (SD); none achieved complete response (CR) or partial response (PR). Furthermore, all patients with amplified genes had PFS of less than six months (*p* < 0.001) (Fig. 2A, B). Similarly, patients with *CCND1* amplification also showed limited responses, with no CR or PR observed (Fig. 2C, D). Furthermore, patients with *ERBB2/CDK12* co-amplification had a more favorable treatment response, with a higher proportion of these patients achieving CR and PR compared to those without such amplifications (n = 36). Additionally, the majority of patients with *ERBB2/CDK12* co-amplification reported a PFS over six months, with only one case exhibiting a PFS of less than six months (*p* = 0.005) (Fig. 2E, F).

**Fig.3:**
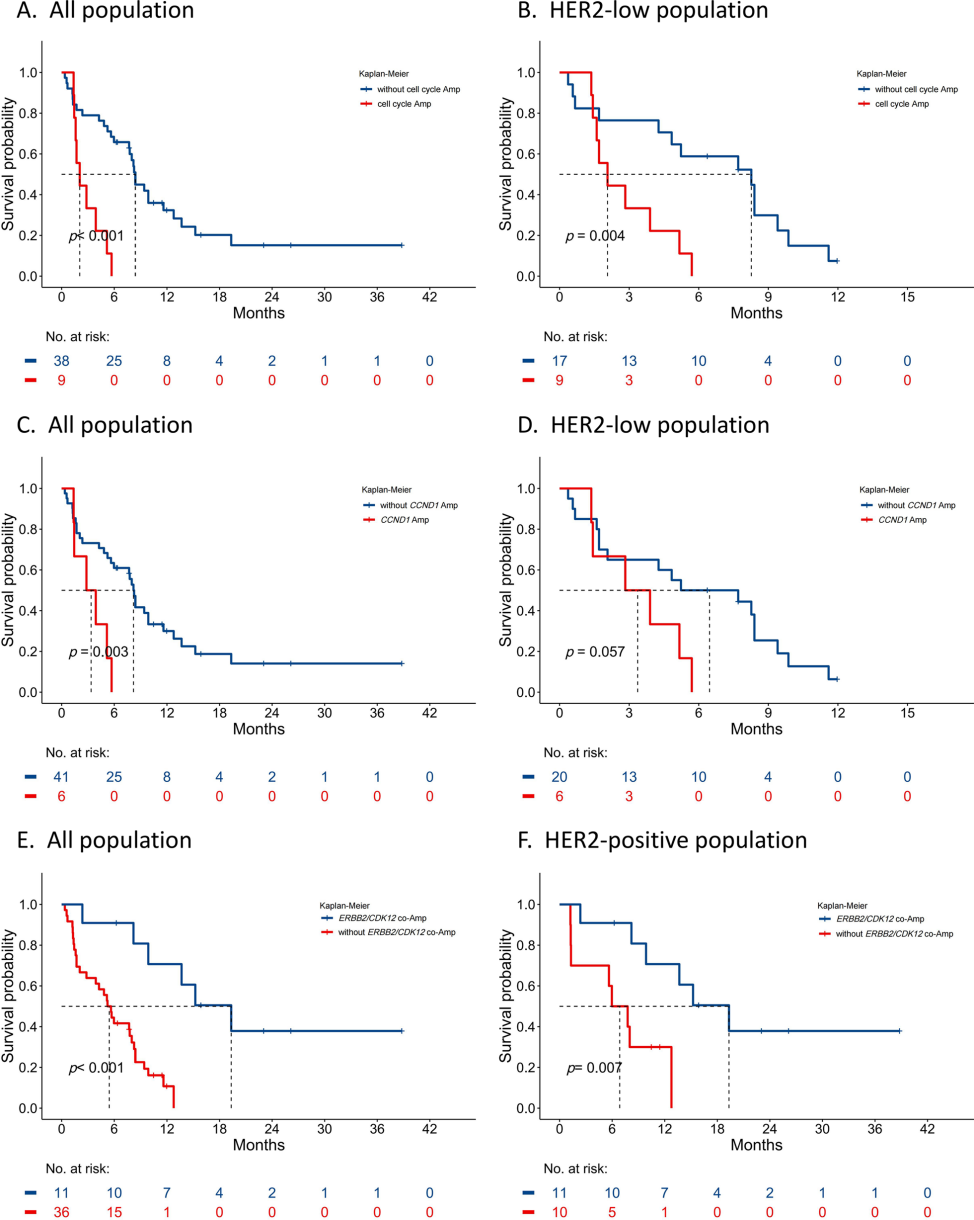
Progression-free survival: **A** All population, stratified by amplification of one or more genes related to the cell cycle pathway; **B** HER2 low population, stratified by amplification of one or more genes related to the cell cycle pathway; **C** All population, stratified by *CCND1* amplification; **D** HER2 low population, stratified by *CCND1* amplification; **E** All population, stratified by *ERBB2/CDK12* co-amplification; and **F** HER2 positive population, stratified by ERBB2/CDK12 co-amplification. Amp, amplification

At the data cut-off, 8 (12.70%) patients were still undergoing treatment, all of whom had achieved a PFS of over six months. The median follow-up time was 19.27 months. Patients with one or more amplifications in cell cycle-related genes showed significantly inferior median PFS than those without these amplifications (2.07 months vs. 8.40 months; HR = 5.24; 95% CI 2.11–13.01; *p* < 0.001) (Fig. 3A). Notably, amplifications in cell cycle-related genes were exclusively identified in HER2-low patients. Subsequent analysis within this subgroup further confirmed that the presence of these amplifications consistently correlated with significantly inferior median PFS (2.07 months vs. 8.27 months; HR = 4.23; 95% CI 1.50–11.91; *p* = 0.004) (Fig. 3B). Patients with *CCDN1* amplification showed the same PFS disadvantages (3.37 months vs. 8.20 months; HR = 3.65; 95% CI 1.39–9.59; *p* = 0.003) (Fig. 3C), while patients with HER2-low disease also had poorer response but did not reach statistical significance (3.37 months vs. 6.47 months; HR = 2.60; 95% CI 0.93–7.27; *p* = 0.057) (Fig. 3D). However, the amplification in cell cycle-related genes and *CCND1* amplification have no significant difference in OS (Fig.S2A, Fig.S2B). Of note, patients enriched for *ERBB2/CDK12* co-amplification achieved longer median PFS (19.33 months vs. 5.43 months; HR = 0.13; 95% CI 0.04–0.45; *p* < 0.001) (Fig. 3E) and further analysis in HER2-positive patients showed that this co-amplification also had a significantly longer PFS (19.33 months vs. 6.87 months; HR = 0.18; 95% CI 0.05–0.72; *p* = 0.007) (Fig. 3F). *ERBB2/CDK12* co-amplification exhibited significant differences in OS both in all population (*p* = 0.002) and in the HER2-positive subgroup (*p* = 0.038) (Fig S2C, Fig. S2D).

**Fig.4:**
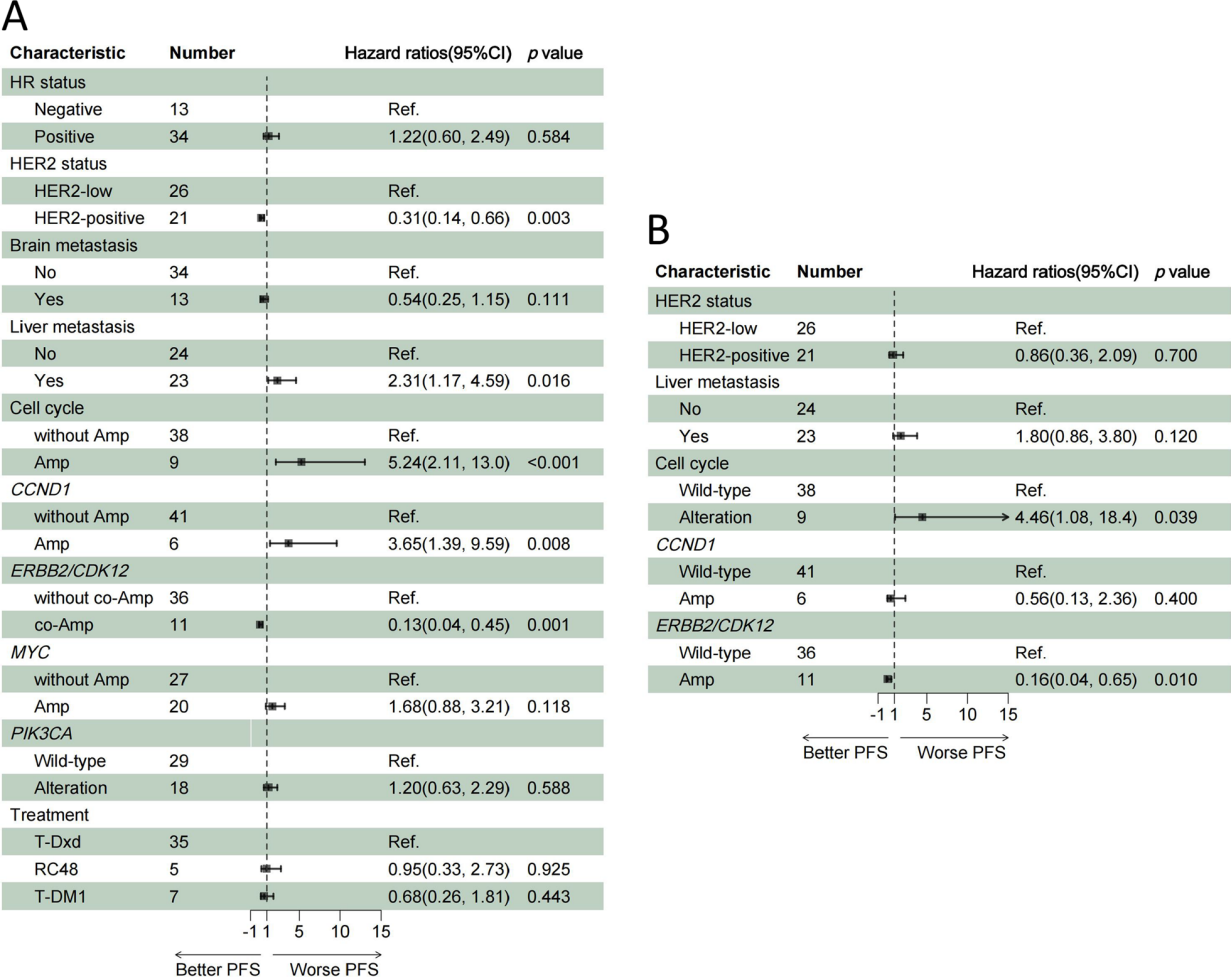
**A** Progression-free survival in univariable Cox regression analysis and **B** Progression-free survival in multivariable Cox regression analysis

To define molecular features that affect the efficacy of HER2 ADCs, we performed univariate and multivariate analyses using the Cox regression hazards model. Among clinical characteristics and genomic alterations, HER2 positive, liver metastasis, amplification in cell cycle-related genes, *CCND1* amplification, *ERBB2/CDK12* co-amplification exhibited notable impacts on PFS (Fig. 4A). In multivariate analysis, detection of amplification in cell cycle-related genes was a significant factor associated with inferior PFS (HR = 4.46; 95% CI 1.08–18.40; *p* = 0.039; Fig. 4B), suggesting that amplification in cell cycle genes may contribute to primary resistance to HER2 ADC. In contrast, patients with *ERBB2/CDK12* co-amplification were independently associated with superior PFS (HR = 0.16; 95% CI 0.04–0.65; *p* = 0.010; Fig. 4B). These findings indicated that *ERBB2/CDK12* co-amplification contributes to more favorable clinical outcomes and could be a therapeutic biomarker for HER2 ADCs.

**Fig.5:**
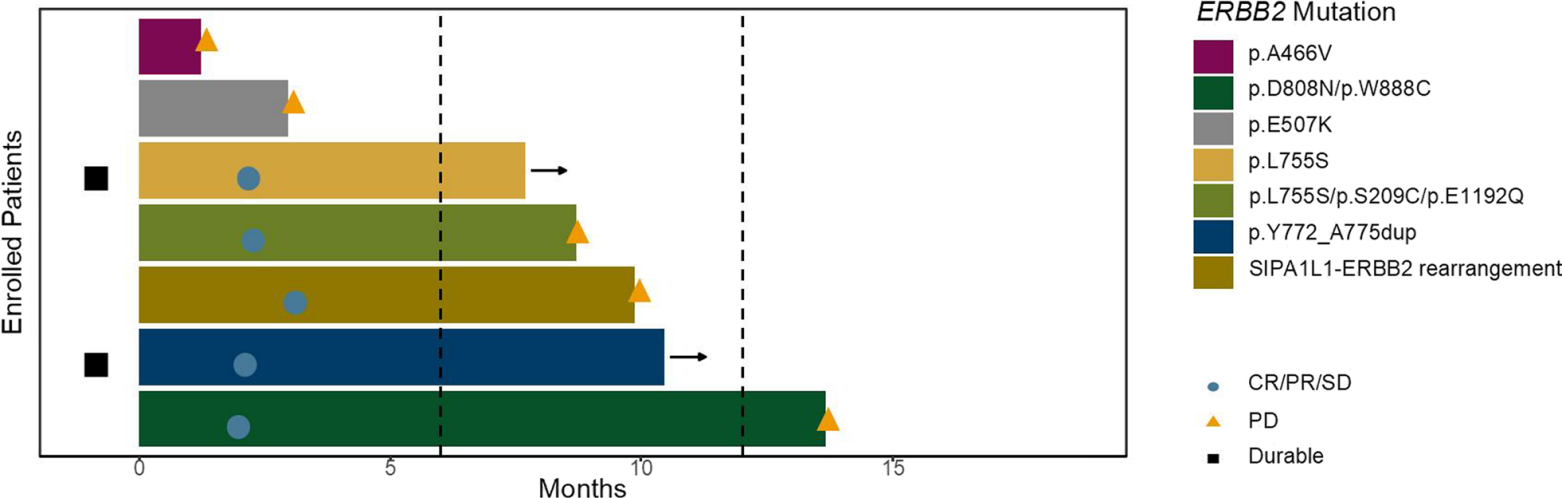
Swimmer plot of the patients with *ERBB2* mutation

Interestingly, in the analysis of the variety of *ERBB2* mutations. p.L755S was the most prevalent, a variant commonly associated with resistance to anti-HER2 therapies [7]. However, two of our patients who received HER2 ADC treatment still achieved PFS of 7.7 months and 8.7 months, and one of them is still receiving treatment (Fig. 5). Additionally, five patients with other mutations also achieved PFS of more than 6 months, and one patient achieved PFS of more than 1 year (Fig. 5).

## Discussion

In the present study, we summarized the genomic data that influence the efficacy of HER2 ADCs in metastatic breast cancer and provided crucial insights into the genomic factors that influence responses to HER2 ADCs, highlighting the potential of precision medicine in this field. To our knowledge, this is the first report to provide a detailed analysis of the genomic alterations that influence the efficacy of HER2 ADCs from real-world data. These findings underscore the pivotal role of genetic testing in refining HER2 ADCs, thereby guiding treatment decisions to improve patient outcomes.

Despite the clinical impact of ADCs, a significant portion of patients exhibit either de novo or primary resistance to these treatments, while others initially respond but eventually develop resistance. Several clinical trials have reported disease progression without any initial tumor response to ADCs in breast cancer [8–10]. Resistance to HER2-targeting ADCs is multifaceted, involving changes in HER2 expression, alterations in intracellular trafficking, impaired lysosomal functions, drug efflux through transporters, and activation of alternative signaling pathways [11]. T-DM1-resistant cells have been found to exhibit decreased levels of *PTEN*, which supports the finding that PI3K/AKT activation contributes to resistance [12]. In the EMILIA trial, patients with absent or decreased tumor PTEN expression derived greater benefit from T-DM1 than capecitabine and lapatinib compared to patients with normal or increased tumor PTEN expression [13]. The potential mutations in signaling pathways that may lead to resistance to HER2 ADCs are diverse. Therefore, it is essential to utilize clinical samples for genetic testing to understand these mutations associated with resistance and improve outcomes for patients.

Cell cycle-related genes play a crucial role in the development of resistance to HER2-targeted therapies in cancer. Alterations in cell cycle-related genes like *CDK2* have been implicated in switching pathway dependence from PI3K/AKT to RAS/MAPK, facilitating resistance to HER2-targeted ADCs [14]. *CDK7* regulates cell cycle progression through transcriptional control rather than direct phosphorylation, making it a promising target in HER2 inhibitor-resistant breast cancer [15]. The cyclin D1-CDK4 axis mediates resistance to HER2-targeted therapy, and Combined HER2-CDK4/6 inhibition synergistically suppresses tumor cell proliferation [16]. In a cohort of 62 HER2-positive breast cancer patients from a clinical trial, higher *CCND1* gene copy number was significantly associated with reduced rates of achieving a pathological complete response to neoadjuvant chemotherapy plus trastuzumab [16]. In our study, we also observed that patients with one or more amplifications in cell cycle-related genes had significantly worse PFS with HER2 ADCs compared to patients without such mutations. Notably, six patients with *CCND1* amplification also had significantly worse PFS. These findings underscore the role of cell cycle-related gene amplifications, particularly *CCND1*, in contributing to resistance and their potential to serve as predictors of poor therapeutic outcomes to HER2 ADCs. However, the relationship between amplification in cell cycle-related genes and HER2 ADC sensitivity still requires further exploration.

Numerous studies have concentrated on identifying predictive biomarkers for the efficacy of HER2 ADCs. Among these, prior research has highlighted that *CDK12* may serve as a predictive marker for the efficacy of these therapies [3]. *CDK12* has been implicated as a pro-tumorigenic factor in breast cancer, primarily due to its co-amplification with the HER2 oncogene and its synergistic interaction with oncogenic pathways such as WNT and IRS1-ErbB-PI3K signaling [17, 18]. This co-amplification is critically associated with resistance to trastuzumab [17] and HER2-targeted tyrosine kinase inhibitors resistance [19]. Beyond its association with HER2 co-amplification, *CDK12* plays a critical biological role in breast cancer progression. It regulates the transcription of DNA damage response (DDR) genes such as *BRCA1* and *FANCI*, which are essential for maintaining genomic stability. Loss or dysregulation of CDK12 leads to impaired DDR and genomic instability, a hallmark of aggressive cancers [20]. Additionally, CDK12 promotes breast cancer stemness and metastasis by activating oncogenic pathways such as c-myc/β-catenin, further driving tumorigenicity and therapy resistance [21]. Interestingly, while CDK12 is often associated with therapy resistance, a prior study showed that *ERBB2/CDK12* co-amplification was a promising positive marker for predicting improved responses to a HER2-targeted bispecific antibody [22]. Consistently, our research also demonstrates that *ERBB2/CDK12* co-amplification significantly prolonged PFS. These results underscore the need for further research to validate the role of *CDK12* and *ERBB2* co-amplification in optimizing HER2 ADCs.

NGS-based assays indicate that HER2 somatic mutations are present in approximately 2–5% of primary breast cancers [23, 24]. HER2 in breast cancer is mainly a point mutation, and common sites of mutation include L755S (86%), V777L (49%), D769H (28%), and S310F (20%) [24]. Some HER2 mutations may induce constitutive HER2 signaling and promote oncogenesis [25]. Clinical results of HER2-targeted therapies in patients with HER2 amplification who harbor HER2 mutations showed a shorter PFS compared with patients without HER2 mutations [26]. The activity of T-DXd in HER2-mutant non-small cell lung cancer (NSCLC) patients has been investigated in the DESTINY-Lung01 trial, a multicenter, international, phase 2 study, showing durable responses in this subset of pretreated patients [27]. However, treatment options are limited for patients with HER2-mutant solid tumors beyond lung cancers. The DESTINY-PanTumor01 phase II basket trial involves patients with unresectable/metastatic solid tumors with HER2 mutations, and recent data from a primary analysis of 20 breast cancer patients showed an encouraging ORR of 50% [28]. Similarly, our study also showed that HER2 ADCs can demonstrate good efficacy in patients with HER2 mutations. Therefore, HER2 ADCs may provide a promising new treatment option for those with HER2-mutated breast cancer.

The limitations of this study lie in its retrospective nature and the heterogeneity in baseline characteristics and treatment factors, which might lead to potential bias. Furthermore, 63 patients who were treated with HER2 ADCs underwent NGS, and more genomic information is needed in the future. Although the current cohort is small, and the data should be interpreted cautiously, the main strength of the present study was that it identified genomic predictors that influence the efficacy of HER2 ADCs in metastatic breast cancer and provided crucial insights into the mechanisms of sensitivity to HER2 ADCs. Therefore, more prospective clinical trials to validate these genomic markers in larger, more diverse patient populations and explore the underlying mechanisms through which these alterations influence therapy resistance and sensitivity are warranted.

In conclusion, our study highlighted the importance of genomic profiling in understanding the differential responses to HER2 ADCs in metastatic breast cancer. The findings suggest that amplifications in cell cycle-related genes may confer primary resistance in HER2-low cancers, while *ERBB2/CDK12* co-amplification appears to be a promising marker for favorable response in HER2-positive breast cancer.

## Supplementary Information

Supplementary material 1: Fig.S1 The overview of alterations in cell cycle-related genes.

Supplementary material 2: Fig.S2 Kaplan-Meier curves for overall survival on the basis of (A) amplification of one or more genes related to the cell cycle pathway in all population, (B) *CCND1* amplification in all population, (C) *ERBB2/CDK12 *co-amplification in all population, and (D) *ERBB2/CDK12* co-amplification in HER2 positive population. Amp, amplification.

Supplementary material 3.

## Data Availability

The datasets generated during the current study are available from the corresponding author upon reasonable request. The key raw data have been uploaded to the Research Data Deposit public platform (https://www.researchdata.org.cn/, RDDA2025425109).
