# Supplementary figures and images for: Impacts of genomic alterations on the efficacy of HER2-targeted antibody–drug conjugates in patients with metastatic breast cancer
Source: J Transl Med. 2025 Jan 13;23:63. doi: 10.1186/s12967-025-06082-5 (PMC11730523; doi:10.1186/s12967-025-06082-5)

Fig.S1

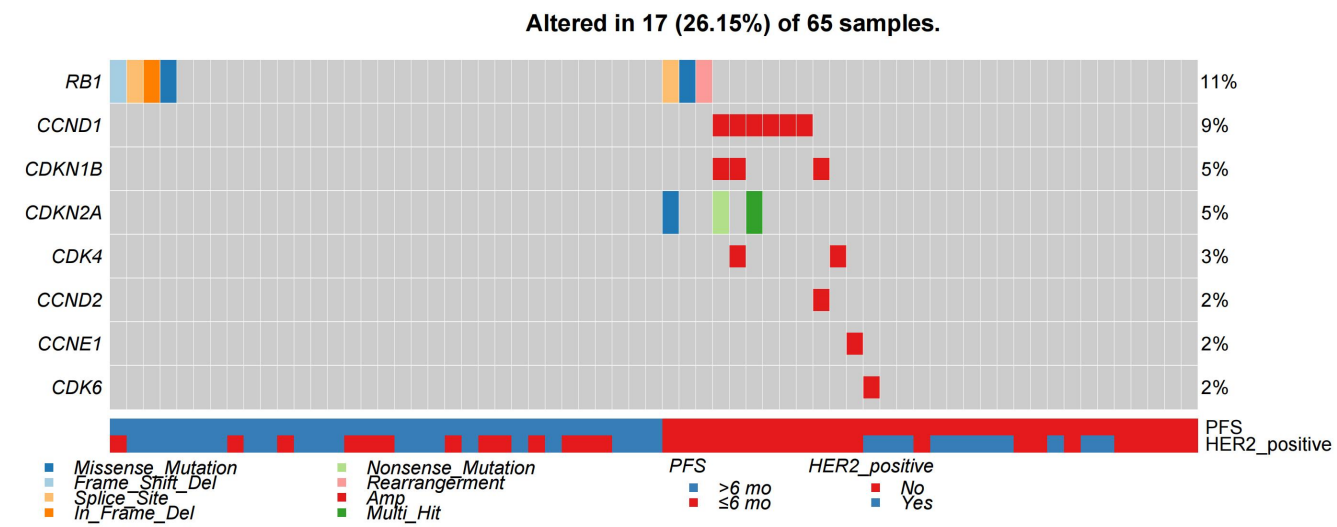

Fig.S1 The overview of alterations in cell cycle-related genes.

Supplement: Supplementary file 1 — Supplementary material 1: Fig.S1 The overview of alterations in cell cycle-related genes. [file 12967_2025_6082_MOESM1_ESM.pdf]
