# Supplementary material for: Impacts of genomic alterations on the efficacy of HER2-targeted antibody–drug conjugates in patients with metastatic breast cancer
Source: J Transl Med. 2025 Jan 13;23:63. doi: 10.1186/s12967-025-06082-5 (PMC11730523; doi:10.1186/s12967-025-06082-5)
Supplement: Supplementary file 2 — Supplementary material 2: Fig.S2 Kaplan-Meier curves for overall survival on the basis of (A) amplification of one or more genes related to the cell cycle pathway in all population, (B) CCND1 amplification in all population, (C) ERBB2/CDK12 co-amplification in all population, and (D) ERBB2/CDK12 co-amplification in HER2 positive population. Amp, amplification. [file 12967_2025_6082_MOESM2_ESM.pdf]

Fig.S2

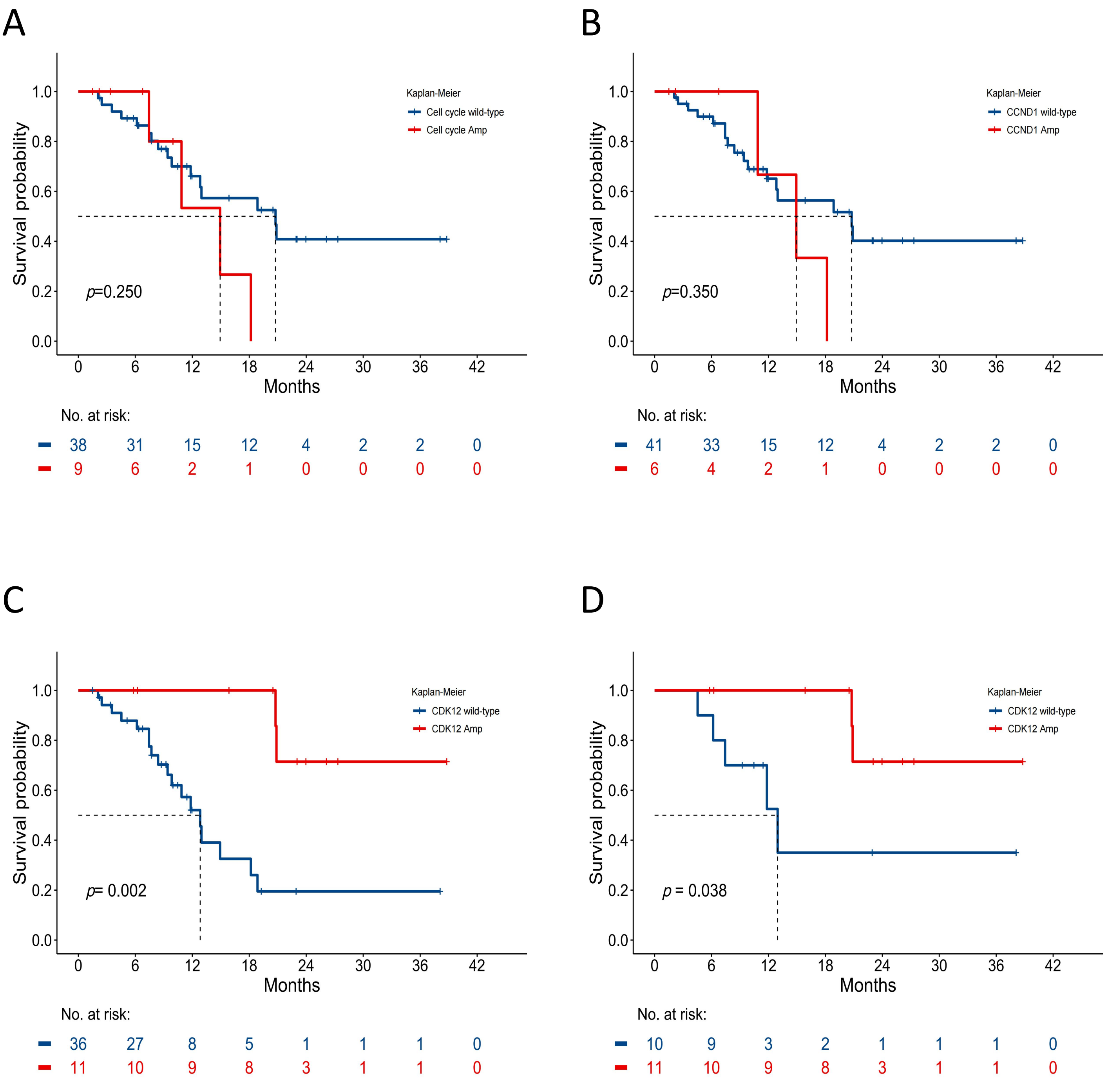

Fig.S2 Kaplan-Meier curves for overall survival on the basis of (A) amplification of one or more genes related to the cell cycle pathway in all population, (B) *CCND1* amplification in all population, (C) *ERBB2/CDK12* co-amplification in all population, and (D) *ERBB2/CDK12* co-amplification in HER2 positive population. Amp, amplification;
